# Genome sequences of two clinical *Escherichia coli* isolates harboring the novel colistin-resistance gene variants *mcr*-*1.26* and *mcr*-*1.27*

**DOI:** 10.1186/s13099-020-00375-4

**Published:** 2020-09-03

**Authors:** Bernd Neumann, Wiebke Rackwitz, Klaus-Peter Hunfeld, Stephan Fuchs, Guido Werner, Yvonne Pfeifer

**Affiliations:** 1 Division of Nosocomial Pathogens and Antibiotic Resistance, Robert Koch Institute, Wernigerode, Germany; 2 Institute for Laboratory Medicine, Microbiology & Infection Control, Northwest Medical Centre, Frankfurt/Main, Germany

**Keywords:** Colistin-resistance, *mcr*-*1*, *Escherichia coli*, IncX4

## Abstract

**Background:**

Colistin is still a widely used antibiotic in veterinary medicine although it is a last-line treatment option for hospitalized patients with infections caused by multidrug-resistant Gram-negative bacteria. Colistin resistance has gained additional importance since the recent emergence of mobile colistin resistance (*mcr*) genes. In the scope of a study on colistin resistance in clinical *Escherichia coli* isolates from human patients in Germany we characterized the *mcr*-*1* gene variants.

**Results:**

Our PCR-based screening for *mcr*-carrying *E. coli* from German patients revealed the presence of *mcr*-*1*-*like* genes in 60 isolates. Subsequent whole-genome sequence-based analyses detected one non-synonymous mutation in the *mcr*-*1* gene for two isolates. The mutations were verified by Sanger sequencing and resulted in amino acid changes Met1Thr (isolate 803-18) and Tyr9Cys (isolate 844-18). Genotyping revealed no relationship between the isolates. The two clinical isolates were assigned to sequence types ST155 (isolate 803-18) and ST69 (isolate 844-18). Both *mcr*-*1* variants were found to be located on IncX4 plasmids of 33 kb size; these plasmids were successfully conjugated into sodium azide resistant *E. coli* J53 Azi^r^ in a broth mating experiment.

**Conclusions:**

Here we present the draft sequences of *E. coli* isolate 803-18 carrying the novel variant *mcr*-*1.26* and isolate 844-14 carrying the novel variant *mcr*-*1.27*. The results highlight the increasing issue of transferable colistin resistance.

## Background

The spread of multidrug-resistant Gram-negative bacteria with resistance to carbapenem antibiotics is a serious threat for public health globally and has led to the reintroduction of colistin, also known as polymyxin E, as a treatment option of last resort [1]. The emergence of colistin resistance in *Escherichia coli* (*E. coli*), a gut commensal of humans and animals, also appearing as opportunistic pathogen, is due to chromosomal mutations or plasmid-mediated genes (*mcr*) that were first described in 2015 [2–4]. So far, a total of 10 different *mcr* genes (*mcr*-*1*–*mcr*-*10*) are known; each gene has its origin in a specific bacterial species [5]. The gene *mcr*-*1* is most prevalent and 25 different *mcr*-*1* variants based on single amino acid substitutions have been submitted to the NCBI database, as of March 2020.

In 2015, the prevalence of colistin resistance in *E. coli* from livestock animals and meat products in Germany was 5–10%; and this colistin resistance was mainly caused by the presence of resistance gene *mcr*-*1* [6]. In contrast, there is no routine testing of colistin susceptibility in human medicine; often only multidrug resistant isolates are occasionally tested. To assess the extent of spread of *mcr*-*1* genes we collected in cooperation with several laboratories, colistin-resistant *E. coli* isolates from human patients in German hospitals over a 4-year-period (2016-2019). MCR-1 producing isolates were identified by PCR screening, and for the isolates described in this study the transferability of *mcr*-*1* genes was tested in broth mate conjugation experiments. Finally, whole-genome sequencing and subsequent in silico analyses were performed. Here, we present the draft genome sequences of *E.* *coli* human isolates 803-18 and 844-18, harboring the novel variants *mcr*-*1.26* and *mcr*-*1.27*, each located on an IncX4 33 kb plasmid.

## Methods

### Bacterial isolates

In 2018, the two colistin-resistant *E. coli* isolates 803-18 and 844-18 were sent from two hospitals in the federal state of Hesse, Germany, to the Robert Koch Institute for confirmation of colistin resistance and identification of the genetic resistance determinant. The *E. coli* isolate no. 803-18 was isolated from blood culture of a 79 years old male patient presenting fever. The second *E. coli* (no. 844-18) was isolated from an intraoperative swab of a 48 years old female patient.

### Phenotypic and PCR-based analyses

In the Robert Koch Institute species identification and antimicrobial susceptibility testing was performed by broth microdilution according to EUCAST (clinical breakpoints (v 10.0) or epidemiological cut-off values (ECOFFs), (http://www.eucast.org)). The following antibiotic substances and substance combinations were tested: ampicillin, cefotaxime, ceftazidime, cefoxitin, meropenem, gentamicin, amikacin, streptomycin, nalidixic acid, ciprofloxacin, chloramphenicol, tetracycline, sulfamethoxazole-trimethoprim and colistin.

PCR screening for the presence of colistin resistance gene *mcr*-*1* and in *E. coli* frequently occurring β-lactamase genes (*bla*_TEM_, *bla*_SHV_, *bla*_CTX-M-groups-1-2-9_) was performed as previously described [4, 7]. Furthermore, a PCR-based method to determine phylogenetic groups of *E. coli* was applied [8].

### Conjugation experiments

The transferability of *mcr*-*1* genes of isolates 803-18 and 844-18 was investigated by broth mate conjugation experiments; the sodium azide-resistant strain *E. coli* J53 Azi^r^ served as the recipient. Transconjugants were selected on Luria–Bertani agar plates containing sodium azide (200 mg/L) and a colistin disk (10 µg). Antimicrobial susceptibilities and presence of *mcr*-*1* and β-lactamase genes were tested for selected transconjugants. To further verify the transfer of plasmids, general plasmid content and plasmid size were determined by S1-nuclease restriction and pulsed-field gel electrophoresis (PFGE) as described before [9].

### Whole-genome sequencing and downstream bioinformatic analyses

DNA extraction was performed using the DNeasy Blood & Tissue kit (Qiagen) and extracted DNA was quantified using the Qubit dsDNA HS Assay Kit (Invitrogen), both according to the manufacturer’s protocols. Genomic libraries were generated with the NexteraXT kit (Illumina). Whole-genome sequencing (WGS) was carried out using the Illumina HiSeq 1500 (2 × 250 bp; HiSeq Rapid SBS Kit v2) benchtop device in ‘Rapid Run Mode’.

Raw reads were processed using the pipeline QCumber (v 2.1.1), where the FastQC (v 0.11.5), Trimmomatic (v 0.36; options ‘sliding window 4:20’, ‘MINLEN: 50 bp’) and Kraken (v 1.0.0) algorithms were included (https://gitlab.com/RKIBioinformaticsPipelines/QCumber/). The draft de novo reconstruction was done using the SPAdes algorithm (v 3.12.0) with default parameters. In a subsequent filtering step, all contigs < 200 bp were excluded. Using the QUAST algorithm without a reference sequence, the quality of draft genome sequences was investigated [10].

The de novo reconstructed sequences were used to extract multilocus sequence types (MLST; Achtmann scheme) and complex types (CT), based on core genome multilocus sequence typing (cgMLST; 2513 allele targets) by utilizing the SeqSphere^+^ software (v 6.0.0, Ridom GmbH) as described before [11, 12]. Gene annotation was determined by the NCBI Prokaryotic Genome Annotation Pipeline (PGAP) [13]. To predict plasmid content in silico, the PlasmidFinder web tool (v 2.1) was used [14]. The NCBI blastn database was used to search for known replicon types, in case of contigs carrying a predicted replicon. Further, the SerotypeFinder (v 2.0) and the VirulenceFinder (v 2.0) web tools were used to characterize the isolates [15, 16].

### Identifying *mcr* genes and variants

Using raw reads, the tool ResFinder (v 3.1.0) was used to identify *mcr* genes [17]. Identified *mcr*-*1*-*like* genes were extracted from the contigs and aligned to a *mcr*-*1.1* reference sequence (gene accession no: NG_050417.1) to calculate a gene-based phylogeny using PhyML (Jukes-Cantor; 500 bootstraps) [4, 18]. Sequences were translated and checked for synonymous and non-synonymous mutations using the Geneious Prime software (v 2020.0.5). To verify identified non-synonymous mutations, primers were designed (Mcr-1a FWD 5′-CAGTATGGGATTGCGCAATGA-3′, Mcr-1a REV 5′-GGGCATTTTGGAGCATGGTC-3′; product size 482 bp, Tm = 59 °C) to perform Sanger sequencing after PCR amplification. The resulting *mcr*-*1*-*like* gene sequences were submitted to NCBI (National Center for Biotechnology Information)/NLM (National Library of Medicine) to determine novel allele numbers (https://www.ncbi.nlm.nih.gov/pathogens/submit-beta-lactamase/) as it has been proposed [19]. Contigs, on which the *mcr*-*1*-*like* genes were located, were investigated by BLAST for known plasmid origins of replication (as of December 2019; https://blast.ncbi.nlm.nih.gov/Blast.cgi).

### Quality assurance

To ensure pure cultures and to phenotypically verify the species, single colonies were repeatedly cultivated on different media (Müller-Hinton agar with sheep blood and Bile-Chrysoidin-Glycerol agar). Further, automated species identification (VITEK 2 GN) was performed. For DNA extraction, single colonies were used. After sequencing, the Kraken algorithm results, also implemented in the QCumber pipeline, were inspected for potential contaminations [20]. De novo assembled genome sequences were quality checked using QUAST.

## Results and discussion

### Antibiotic resistance and *mcr*-*1* transferability

Both *E. coli* isolates 803-18 and 844-18 were resistant to colistin (MIC = 4 mg/L), ampicillin, sulfamethoxazole/trimethoprim, nalidixic acid, ciprofloxacin and tetracycline (Table 1). Isolate 803-18 was additionally resistant to streptomycin, and isolate 844-18 was additionally resistant to chloramphenicol. Both isolates remained fully susceptible to cephalosporins and carbapenems (Table 2).

**Table 1:** Typing results and gene detections of the *E. coli* strains 803-18 and 844-18

Strain no.	Genotyping/phylogeny			Resistance gene detection		Antibiotic susceptibility testing	Plasmid content analyses		Virulence gene detection
	*E. coli* phylogenetic group^a^	Multilocus sequence type (ST)^b^	complex type (CT)^b^	Resistance genes PCR	Resistance genes ResFinder^c^	Resistance to antibiotics^d^	Replicon prediction with PlasmidFinder^c^	Plasmid sizes S1-PFGE^e^	Virulence gene {protein function} VirulenceFinder^c^
803-18	B1	ST155	CT7500	*bla*_TEM_, *mcr*-*1*-*like*	*bla*_TEM-1B_, *mcr*-*1*-*like, aadA17*-*like, strA*-*like,strB*-*like, tetA, sul2, dfrA14*-*like, lnuF*	AMP, PIP, CST, STR^f^, NAL^f^, MOX, CIP, TET^f^, SXT	IncFIC(FII), IncFIB(AP001918), IncI1, **IncX4**, ColRNAI, Col(MG828)	33 kb, 90 kb, 100 kb	gad{glutamate decarboxylase}, iroN{enterobactin siderophore receptor protein}, iss{increased serum survival}, lpfA{long polar fimbriae}
844-18	D	ST69	CT7508	*bla*_TEM_, *mcr*-*1*-*like*	*bla*_TEM-1B_, *mcr*-*1*-*like, aadA1, mph(B), dfrA1, tetA, sul1, catA1*-*like*	AMP, PIP, CST, NAL^f^, MOX, CIP, TET^f^, CMP, SXT	IncFII, IncFIB(AP001918), **IncX4**, Col(BS512), ColRNAI, Col156, Col(MG828)	33 kb, 80 kb, 160 kb	air{enteroaggregative immunoglobulin repeat protein}, cma{colicin M}, eilA{Salmonella HilA homolog}, gad{glutamate decarboxylase}, iroN{enterobactin siderophore receptor protein}, iss{increased serum survival}, lpfA{long polar fimbriae}

^a^PCR according to Clermont et al. [8]; ^b^information extracted from whole-genome sequence (WGS) data (Illumina, HiSeq), using the SeqSphere^+^ software suite (v 6.0.0) with integrated MLST and cgMLST schemes (https://enterobase.warwick.ac.uk/species/index/ecoli; http://www.ridom.de/seqsphere/u/Task_Template_Sphere.html); ^c^information extracted from WGS data, ResFinder (v 3.2)/PlasmidFinder (v 2.1)/VirulenceFinder (v 2.0) (http://www.genomicepidemiology.org/); ^d^Broth microdilution and automated testing (VITEK 2, card AST N248) according to EUCAST (v 10.0); abbreviations of antibiotics: ampicillin (AMP), piperacillin (PIP), colistin (COL), sulfamethoxaole-trimethoprim (SXT), nalidixic acid (NAL), moxifloxacin (MOX), ciprofloxacin (CIP), oxytetracycline (OTE), streptomycin (STR); ^e^S1-nuclease restriction of whole genomic DNA and pulsed-field gel electrophoresis (PFGE) according to Barton et al. [9]; ^f^for these substance only epidemiological cut-off values (ECOFFs) are available to separates microorganisms without (wild type) and with acquired resistance mechanisms (non-wild type) to the agent in question (http://www.eucast.org); In bold print: plasmid replicon type (incompatibility group) of plasmids that carry the colistin resistance gene *mcr*-*1*-*like*

**Table 2:** Antibiotic susceptibilities of *mcr*-*1*-*like* positive donor strains and transconjugants (MICs in mg/L)

Strain no.	AMP	CTX	CAZ	FOX^b^	GEN	AMK	STR^b^	CMP	TET^b^	NAL^b^	CIP	MER	TRS	COL
803/18 *E. coli* (B1, ST155)^a^	> 16	≤ 1	≤ 2	4	1	≤ 2	> 64	8	> 8	> 32	0.5	≤ 0.063	> 128	4
803/18 Tc1 *E. coli* J53 Azi^r^ (A, ST10)^a^	> 16	≤ 1	≤ 2	8	4	16	> 64	≤ 4	1	16	≤ 0.063	≤ 0.063	≤ 4	2
844/18 *E. coli* (D, ST69)^a^	> 16	≤ 1	≤ 2	8	2	4	16	> 32	>8	> 32	0.5	≤ 0.063	> 128	4
844/18 Tc1 *E. coli* J53 Azi^r^ (A, ST10)^a^	4	≤ 1	≤ 2	4	≤ 0.5	≤ 2	≤ 4	32	>8	8	≤ 0.063	0.125	> 128	2
*E. coli* J53 Azi^r^ (A, ST10)^a^ recipient	4	≤ 1	≤ 2	2	≤ 0.5	≤ 2	≤ 4	8	1	8	≤ 0.063	≤ 0.063	≤ 4	0.125

Antibiotic susceptibility testing was performed by broth microdilution with MIC interpretation of minimum inhibitory concentrations (MICs) according recommendations of the European Committee on Antimicrobial Susceptibility testing (EUCAST v 10.0). Tc1, transconjugants; ^a^*E. coli* phylogentic group determined by PCR according Clermont et al. [8], and multilocus sequence type (ST) according to the MLST scheme of Achtmann (Wirth et al. [12]). ^b^For these substance only epidemiological cut-off values (ECOFFs) are available to separates microorganisms without (wild type) and with acquired resistance mechanisms (non-wild type) to the agent in question (http://www.eucast.org)

*AMP* ampicillin, *CTX* cefotaxime, *CAZ* ceftazidime, *FOX* cefoxitin, *GEN* gentamicin, *AMK* amikacin, *STR* streptomycin, *NAL* nalidixic acid, *CMP* chloramphenicol, *TET* tetracycline, *CIP* ciprofloxacin, *MER* meropenem, *TRS* trimethoprim/sulfamethoxazole, *COL* colistin

The PCR-confirmed *mcr*-*1*-*like* genes in both isolates could be transferred in conjugation experiments. Transconjugant 803-18 Tc1 harbored two plasmids (ca. 33 kb and 90 kb) and showed a colistin MIC of 2 mg/L. Additional resistance to streptomycin and ampicillin was detected; presence of *mcr*-*1*-*like* and β-lactamase gene *bla*_TEM_ was confirmed by PCR. Transconjugant 844-18 Tc1 was positive for the *mcr*-*1*-*like* gene, showed a colistin MIC of 2 mg/L and harbored one plasmid of ca. 33 kb size. Additional resistance to chloramphenicol, tetracycline and sulfamethoxazole-trimethoprim was detected (Table 2). These resistances might be encoded on smaller plasmids but plasmids smaller than 20 kb were not detectable by S1-PFGE.

### General genome features of *E. coli* isolates 803-18 and 844-18

A total of 1,349,261 raw reads were obtained for *E. coli* no. 803-18 and 1,700,507 for *E. coli* no. 844-18. After de novo reconstruction of isolate 803-18, 154 scaffolds (155 contigs) were assembled, with N50: 119,280 bp and L50: 13. On average, the assembled draft genome was covered 84x. The draft genome size was determined as 4.92 Mb, with 50.6% GC content; and 4854 genes, encoding 4516 proteins, were predicted. The draft assembly of isolate 844-18 resulted in 192 scaffolds (193 contigs), with N50: 145,421 bp and L50: 12; with 45 × genome coverage. The determined draft genome size was 5.31 Mb, with 50.6% GC content; and 5228 genes, encoding 4923 proteins, were predicted.

### Resistance and virulence gene predictions

ResFinder detected the presence of several resistance genes in isolates 803-18 and 844-18, respectively (Table 1) contributing to resistance to colistin (*mcr*-*1*-*like*), penicillins (*bla*_TEM-1B_), sulfonamides (*sul1, sul2*), trimethoprim (*dfrA1, dfrA14*-*like*), aminoglycosides (*str*-*A*-*like, str*-*B*-*like, aadA1*), tetracyclines (*tetA*) and phenicols (*catA1*-*like*) (Table 1). These results corresponded to the phenotype of the isolates, which highlights the general applicability of WGS-based data also for antibiotic resistance predictions, as it was discussed before [21].

VirulenceFinder detected genes in both isolates that were associated with fitness or virulence traits (colonization and fitness factors) in *E. coli,* named *iroN, gad, lpfA* and *iss* encoding enterobactin siderophore receptor protein, glutamate decarboxylase, long polar fimbriae and increased serum survival, respectively (Table 1). For isolate 844-18 three further genes were detected: *cma,* encoding the bacteriocin colicin M, *air* encoding the adhesin enteroaggregative immunoglobulin repeat protein and its regulator *eilA* (*hilA* homolog in *Salmonella*) [22, 23]. However, virulence genes (e.g*. eae* and *stx*) that are associated with a specific pathotype (e.g. EAEC and EHEC) were not detected in the two isolates.

### WGS-based typing

The different typing approaches showed that the two isolates were genotypically dissimilar; at core-genome level (cgMLST-analysis) the isolates showed a distance of 2362 alleles to each other. Isolate 803-18 was assigned to phylogenetic group B1, serotype H45, sequence type ST155 and cgMLST-based complex type CT7500; isolate 844-18 was identified as phylogenetic group D, serotype O15:H18, ST69 and CT7508 (Table 1). Phylogenetic group B1 is known to mainly comprise environmental and animal isolates, whereas phylogenetic group D is known to include more (urogenital-) pathogenic *E. coli* [24]. This result seems to be concordant with MLST, since *E. coli*-ST155 has been described as sequence type with zoonotic potential and plasmid-mediated spread of antibiotic resistance, whereas *E. coli*-ST69 was described as a pandemic and pathogenic lineage [25, 26]. The latter is supported by the presence of additional virulence genes in *E. coli*-ST69 isolate 844-18 that are involved in adherence to epithelial cells and biofilm formation (adhesion AIR and regulator protein EilA), and the fitness factor colicin M, a bacteriocin that kills other sensitive *E. coli* strains [22, 23]. The typing results of the two isolates, led to the assumption that there is no certain Mcr-1-like producing strain in the hospitals, instead this might be a hint for a potentially community-based influx of *mcr*-*1*-*like* mediated colistin-resistance via different strains into hospitals, as discussed in other studies [27, 28].

### Plasmid content

Based on the PlasmidFinder results, for both isolates several plasmids could be predicted; and S1-PFGE analysis confirmed the presence of at least three plasmids in each of the two isolates (Table 1). PlasmidFinder was able to predict more plasmids, e.g. several Col-like plasmids (Table 1). These plasmids of small size could not be seen in S1-PFGE analysis and were not further analyzed in the present study.

Blast analyses of the scaffolds carrying the *mcr*-*1*-*like* genes predicted their location on IncX4 plasmids, with high similarity (> 99%) to *mcr*-*1* of an IncX4 plasmid of 33 kb size (GenBank accession: CP042970.1) of an *E. coli* isolate from raw milk cheese in Egypt. For our isolates, the IncX4 plasmid could be reconstructed with two scaffolds: for isolate 803-18 a 32,744 bp *mcr*-*1*-*like*-positive scaffold and an 820 bp scaffold; for isolate 844-18 a 32,738 bp *mcr*-*1*-*like*-positive scaffold and an 821 bp scaffold. However, in CP042970.1 and in both reconstructed IncX4 plasmids the *mcr*-*1*-*like* gene was not part of the IS*Apl1* transposon that is known to be associated with *mcr*-*1* dissemination as described previously [29, 30]. Instead both plasmids included the IS*6*-*like* element (encoding an IS*26* family transposase). We reconstructed a 33 kb ring structure, with 100% coverage and 99.6% pairwise identity compared to plasmid CP042970.1. Furthermore, high identity with further *mcr*-*1* carrying plasmids in the NCBI database was detected (GenBank accession: MF449287.1; MK172815.1). These were from fresh water from Italy (MF449287.1) and human origin from Russia (MK172815.1), also showing > 99% coverage and > 99% pairwise identity with the reconstructed 33 kb plasmid of 803-18 and 844-18. This indicates a worldwide spread of this type of plasmid with colistin resistance gene *mcr*-*1* in *E. coli.*

### Analyses of *mcr*-*1*-*like* genes

**Fig. 1:**
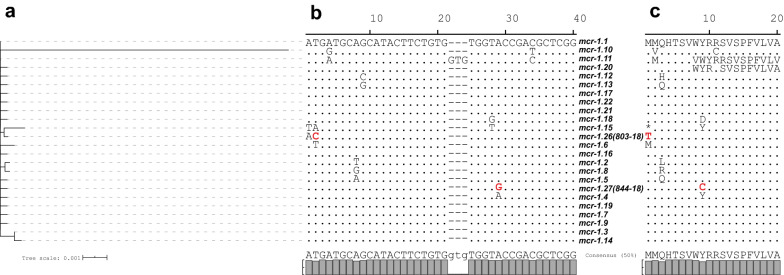
Relatedness of *mcr*-*1* variants and characteristics of novel identified *mcr*-*1.26* and *mcr*-*1.27*. The image visualizes the relatedness of different *mcr*-*1* allele variants, including the two novel ones described in this study (*mcr*-*1.26* and *mcr*-*1.27*), as single-gene phylogenetic tree (**a**). Further, an excerpt of the first 40 bp of *mcr*-*1* genes were displayed and with bold and red letters the novel properties of *mcr*-*1.26* and *mcr*-*1.27* gene sequences are highlighted (**b**). In **c**, the first 20 amino acids of translated *mcr*-*1* genes were shown and with bold and red letters the non-synonymous changes of *mcr*-*1.26* and *mcr*-*1.27* are highlighted. Visualization was realized using iTOL (v 5) [32]

Alignment of the extracted *mcr*-*1*-*like* genes of isolates 803-18 and 844-18 and known *mcr*-*1* variants (as of December 2019) to the reference sequence of *mcr*-*1.1* (NG_050417.1) revealed putative point mutations (Fig. 1). These point mutations were confirmed by PCR amplification and Sanger sequencing. Subsequent translation revealed these point mutations were non-synonymous mutations, resulting in amino acid substitutions Met1Thr (isolate 803-18) and Tyr9Cys (isolate 844-18) (Fig. 1B). The substitution Met1Thr in isolate 803-18 was due to the ACG (Thr) codon that has been reported by Hecht et al. for its potential role in non-canonical initiation in *E. coli* [31]. It is important to note that in *mcr*-*1.26* an ATG (Met) is present immediately after ACG (Thr) and therefore we are uncertain of the actual effect of Met1Thr on the translation initiation of *mcr*-*1.26* in isolate 803-18. This warrants further investigation. However, the conjugation experiment confirmed an increase in colistin MIC of the transconjugant (2 mg/L, Table 2).

Both *mcr*-*1*-*like* sequences were submitted to NCBI/NLM and assigned with two novel *mcr*-*1* allele numbers: *mcr*-*1.26* (isolate 803-18; NCBI Reference Sequence: NG_068217.1; RefSeq CDS region in nucleotide: JAAGSA010000042.1 3574-5196(+); protein accession: WP_034169413.1) and *mcr*-*1.27* (isolate 844-18; NCBI Reference Sequence: NG_068218.1; RefSeq CDS region in nucleotide: JAAGSB010000042.1 27547-29172(−); protein accession: WP_163397051.1). The identification of two novel *mcr*-variants in hospitals in the same region and within 1 year shows that the spread of plasmid-mediated colistin-resistance seems to rapidly progress and new variants are constantly emerging [28].

## Conclusions

Through collections and analysis of colistin-resistant *E. coli* from clinical samples two novel *mcr*-*1* variants were identified, named *mcr*-*1.26* and *mcr*-*1.27*. The IncX4 plasmids that carried these *mcr*-*1* variants were 99.6% identical to previously described plasmids in *E. coli* from livestock and food samples. This raises the possibility that there might be a ‘plasmid reservoir’ outside hospital environments. However, the likelihood of an established plasmid clone circulating in the hospital can also not be excluded because both *mcr*-*1* variants were identified on the widely disseminated Incx4 plasmids that are known for harboring *mcr*-*1* genes. Further, these plasmids were found in two different *E. coli* isolates (ST155 and ST69) with the latter being described as one pandemic lineage circulating in hospitals. Future genome-based surveillance studies of large scale would help elucidating putatively plasmid-associated transmissions of *mcr*-*1.*

## Data Availability

Raw reads, as well as de novo assembled draft genome sequences of the sequenced *E.* *coli* isolates of this study (n = 2) were submitted to GenBank and the Sequence Read Archive database of the National Center for Biotechnology Information (NCBI) and are available under BioProject accession PRJNA605141 (https://www.ncbi.nlm.nih.gov/bioproject/PRJNA605141). The novel variants *mcr*-*1.26* and *mcr*-*1.27* were available under BioProject accession PRJNA313047 (https://www.ncbi.nlm.nih.gov/bioproject/PRJNA313047), with NCBI Reference Sequence: NG_068217.1 (*mcr*-*1.26*) and NG_068218.1 (*mcr*-*1.27*).

## Abbreviations

CDS: Coding sequence
cgMLST: Core genome multilocus sequence typing
CT: Complex type
ECOFFs: Epidemiological cut-off values
PFGE: Pulsed-field gel electrophoresis
SNP: Single nucleotide polymorphism
ST: Sequence type
WGS: Whole-genome sequencing
